# Combined treatment of an aortosplenic bypass followed by coil embolization in the treatment of pancreaticoduodenal artery aneurysms caused by median arcuate ligament compression: a report of two cases

**DOI:** 10.1186/s40792-021-01260-1

**Published:** 2021-08-04

**Authors:** Shuhei Kii, Hirofumi Kamachi, Daisuke Abo, Takuya Kato, Yousuke Tsuruga, Kenji Wakayama, Tatsuhiko Kakisaka, Takeshi Soyama, Toshiya Kamiyama, Tomonori Ooka, Satoru Wakasa, Akinobu Taketomi

**Affiliations:** 1 Department of Gastroenterological Surgery I, Hokkaido University Graduate School of Medicine, N15, W7, Kita-ku, Sapporo, 060-8638 Japan; 2 Department of Diagnostic and Interventional Radiology, Hokkaido University Hospital, N15, W7, Kita-ku, Sapporo, 060-8638 Japan; 3 Department of Cardiovascular and Thoracic Surgery, Hokkaido University Faculty and School of Medicine, N15, W7, Kita-ku, Sapporo, 060-8638 Japan

**Keywords:** Bypass surgery, Embolization, Pancreaticoduodenal artery aneurysm

## Abstract

**Background:**

Pancreaticoduodenal artery aneurysms (PDAAs) are rare visceral aneurysms, and prompt intervention/treatment of all PDAAs is recommended at the time of diagnosis to avoid rupture of aneurysms. Herein, we report two cases of PDAA caused by the median arcuate ligament syndrome, treated with surgical revascularization by aortosplenic bypass followed by coil embolization.

**Case presentation:**

*Case 1* A 54-year-old woman presented with a chief complaint of severe epigastralgia and was diagnosed with two large fusiform inferior PDAAs and celiac axis occlusion. To preserve the blood flow of the pancreatic head, duodenum, liver, and spleen, we performed elective surgery to release the MAL along with aortosplenic bypass. At 6 days postoperatively, transcatheter arterial embolization was performed. At the 8-year 6-month follow-up observation, no recurrent perfusion of the embolized PDAAs or rupture had occurred, including the non-embolized small PDAA, and the bypass graft had excellent patency.

*Case 2* A 39-year-old man who had been in good health was found to have a PDAA with celiac stenosis during a medical checkup. Computed tomography and superior mesenteric arteriography showed severe celiac axis stenosis and a markedly dilated pancreatic arcade with a large saccular PDAA. To preserve the blood flow of the pancreatic arcade, we performed elective surgery to release the MAL along with aortosplenic bypass. At 9 days postoperatively, transcatheter arterial embolization was performed. At the 6-year 7-month follow-up observation, no recurrent perfusion or rupture of the PDAA had occurred, and the bypass graft had excellent patency.

**Conclusion:**

Combined treatment with bypass surgery and coil embolization can be an effective option for the treatment of PDAAs associated with celiac axis occlusion or severe stenosis.

## Introduction

Pancreaticoduodenal artery aneurysms (PDAAs) are rare, accounting for about 2% of all visceral aneurysms [1]. PDAAs are frequently associated with celiac arterial occlusion or stenosis due to compression by the median arcuate ligament (MAL) and atherosclerosis. We herein report two cases of combined treatment with bypass surgery and coil embolization for PDAAs associated with celiac axis occlusion or stenosis due to compression by the MAL.

## Case presentation

### Case 1

**Fig. 1:**
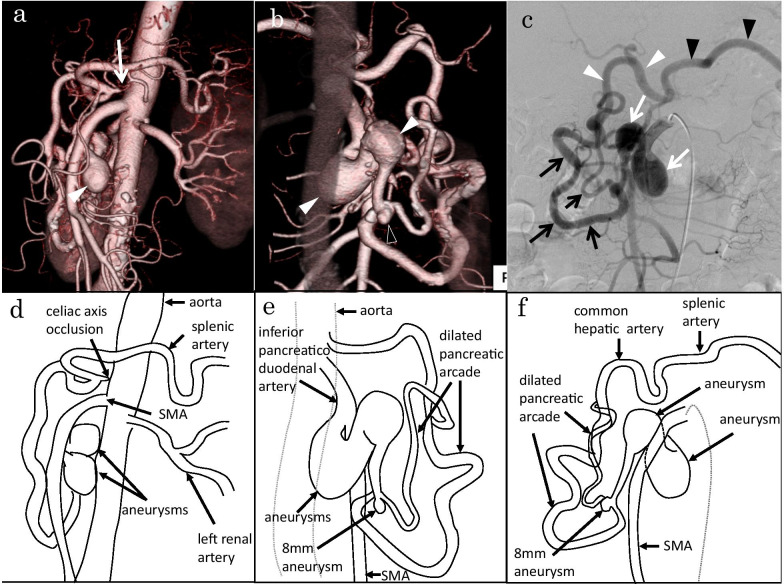
Images before bypass surgery from Case 1. **a**, **b** Three-dimensional computed tomogram showing celiac axis occlusion (white arrow) and a markedly dilated pancreatic arcade with two large fusiform inferior pancreaticoduodenal artery aneurysms (PDAAs) (white arrowheads) a small PDAA (transparent arrowhead). **c** Angiogram of the superior mesenteric artery showing a markedly dilated pancreatic arcade (black arrows) with two large fusiform inferior PDAAs (white arrows) and retrograde flow of the gastroduodenal artery and common hepatic artery via the pancreatic arcade (white arrowheads) and retrograde flow of splenic artery via the common hepatic artery (black arrowheads). **d** The schematic diagram of **a**. **e** The schematic diagram of **b**. **f** The schematic diagram of **c**

A 54-year-old woman presented with a chief complaint of severe epigastralgia. She had no history of pancreatitis, abdominal trauma, or connective tissue disorders. A computed tomography (CT) scan and angiography of the superior mesenteric artery (SMA) showed celiac axis occlusion and a markedly dilated pancreatic arcade with two large fusiform inferior PDAAs (Fig. 1a–f). The respective long diameters of the proximal and distal aneurysms of the inferior pancreaticoduodenal artery (PDA) were 21 mm and 19 mm. A small PDAA measuring 8 mm was noted in the bifurcation of the anterior and posterior branches. Angiography of the SMA showed retrograde flow of the gastroduodenal artery and common hepatic artery via the pancreatic arcade. The splenic artery was perfused via the common hepatic artery. We could not perform angiography of celiac artery because of celiac axis occlusion. No extravasation or hematoma formation was present. Celiac axis occlusion was speculated to have occurred secondary to compression by the MAL.

Transcatheter arterial embolization (TAE) was considered as the first treatment option. However, we hesitated to perform TAE because (1) coil packing was necessary for complete embolization of these fusiform PDAAs, and (2) complete embolization of the PDAAs was considered highly likely to result in marked diminishment of blood flow of the pancreatic head, duodenum, liver, and spleen because of the celiac axis occlusion. In addition, the aneurysm was located around the pancreatic head. Surgical treatment would require pancreaticoduodenectomy with arterial bypass for revascularization of the celiac territory, but this would have been highly invasive.

**Fig. 2:**
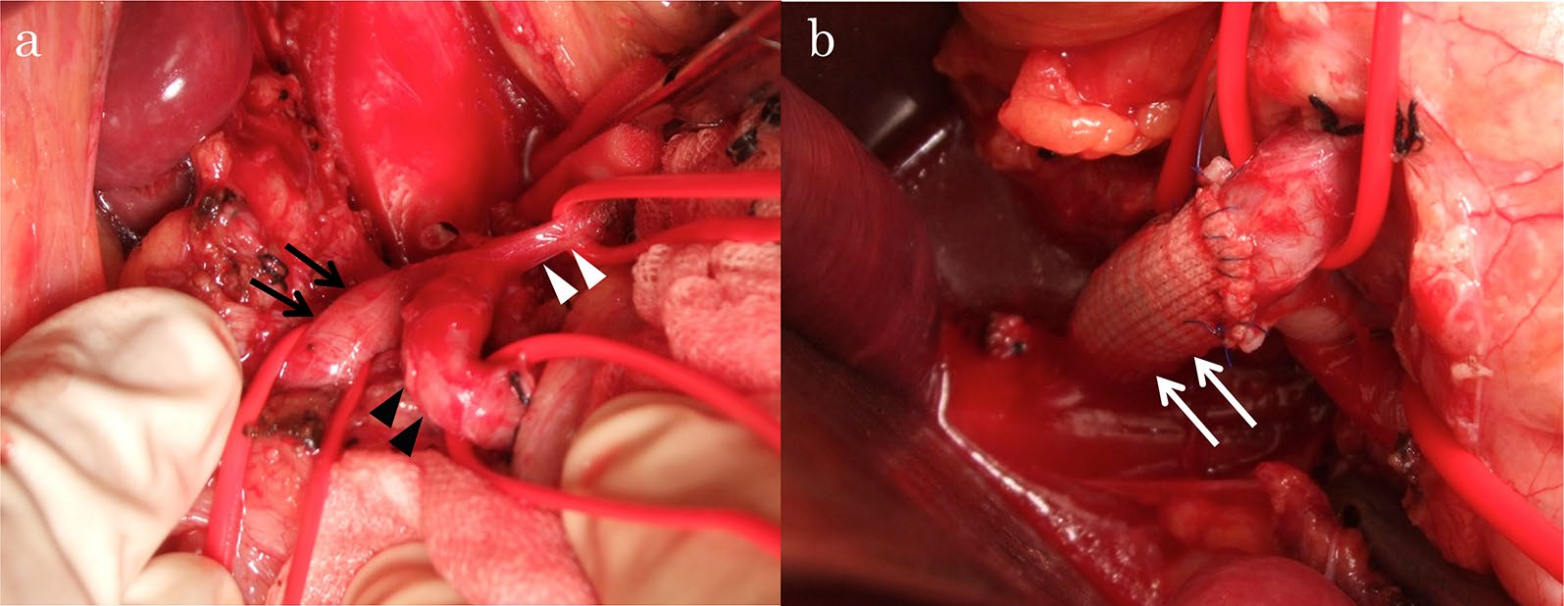
Intraoperative findings of Case 1. **a** After release of the median arcuate ligament, the blood flow of the celiac axis (white arrowhead) was not restored. The blood supply of the common hepatic artery (black arrow) and splenic artery (black arrowheads) arose from retrograde blood flow. **b** Aortosplenic bypass (white arrow) was performed

Therefore, we performed elective surgery to restore the normal arterial flow of the celiac axis. Intraoperatively, the general exploration findings were normal, and no hematoma was present. After the MAL release, the blood flow of the celiac axis was not restored (Fig. 2a). Therefore, aortosplenic bypass was performed (Fig. 2b). An 8-mm ringed Dacron graft (Gelsoft ERS straight 8 mm; Terumo, Tokyo, Japan) was anastomosed side-to-end to the abdominal aorta and side-to-end to the splenic artery.

**Fig. 3:**
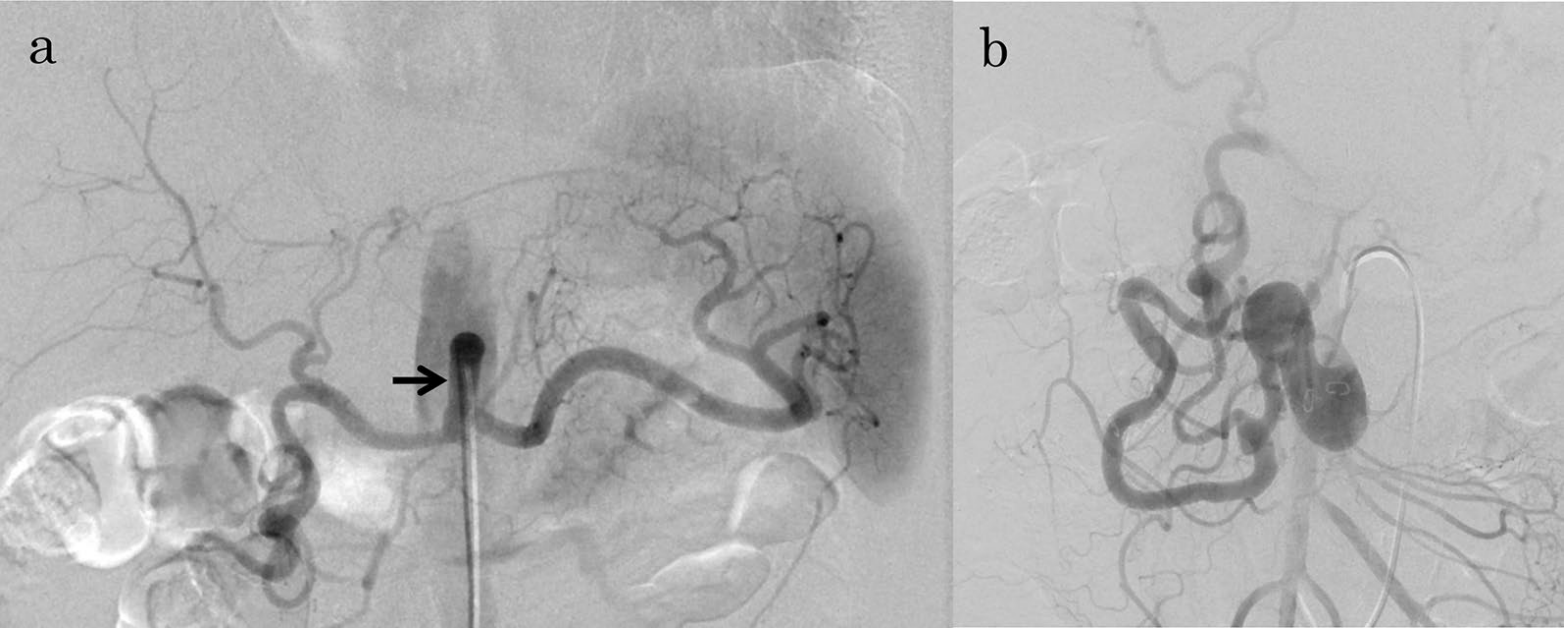
Images after bypass surgery from Case 1. **a**, **b** Angiogram via bypass and superior mesenteric artery performed 6 days after bypass surgery showing excellent patency of the bypass (black arrow) and antegrade flow of the hepatic and splenic arteries and decreased retrograde flow of the PDA via the superior mesenteric artery

At 6 days postoperatively, a CT scan and angiography via bypass (Fig. 3a) and SMA (Fig. 3b) showed excellent patency of the bypass and antegrade flow of the hepatic and splenic arteries via the bypass and decreased retrograde flow of the PDA via the SMA, compared with the preoperative angiography (Fig. 1c).

**Fig. 4:**
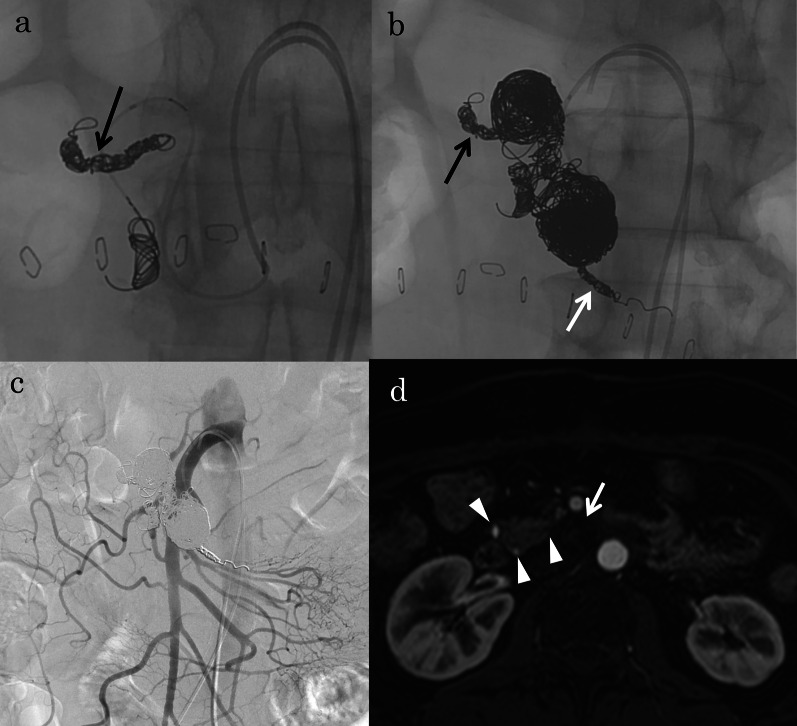
Images from Case 1. **a, b** Fluoroscopic images during coil embolization showing embolization of the inferior pancreaticoduodenal artery (PDA) combined with packing of the aneurysms. Additional embolization with coils is shown in the branch of the inferior PDA (black arrows) and first jejunal artery (white arrow) arising from the proximal inferior PDA to prevent reperfusion of the PDA aneurysms (PDAAs). **c** Post-embolization angiogram via the superior mesenteric artery showing complete occlusion of the aneurysms. **d** Contrast-enhanced magnetic resonance imaging after treatment showing disappearance of the arterial flow in the proximal PDAA (white arrow) and decreased thickness of the pancreatic arcades (white arrowheads)

Therefore, TAE of the two inferior PDAAs was performed. Proximal and distal embolization of the inferior PDA combined with packing of the aneurysms was performed (Fig. 4a, b). In total, 29 detachable and 28 pushable microcoils were used. Post-embolization angiography showed complete occlusion of the aneurysms with excellent preservation of the non-embolized pancreatic arcade (Fig. 4c). Twelve days postoperatively, she had a stomachache and nausea and abdominal CT showed dilation of stomach and duodenum, so the symptoms were thought to be a consequence of duodenal ischemia. She underwent conservative treatment such as nasogastric tube decompression, intravenous fluids, bowel rest and the symptoms were improved in a week. She was administered 100 mg of aspirin daily after surgery and was discharged in good condition on postoperative day 30. At the 8-year 6-month follow-up observation, no recurrent perfusion of the embolized PDAAs (Fig. 4d) or rupture, including the non-embolized small PDAA, had occurred. The bypass graft showed excellent patency.

### Case 2

**Fig. 5:**
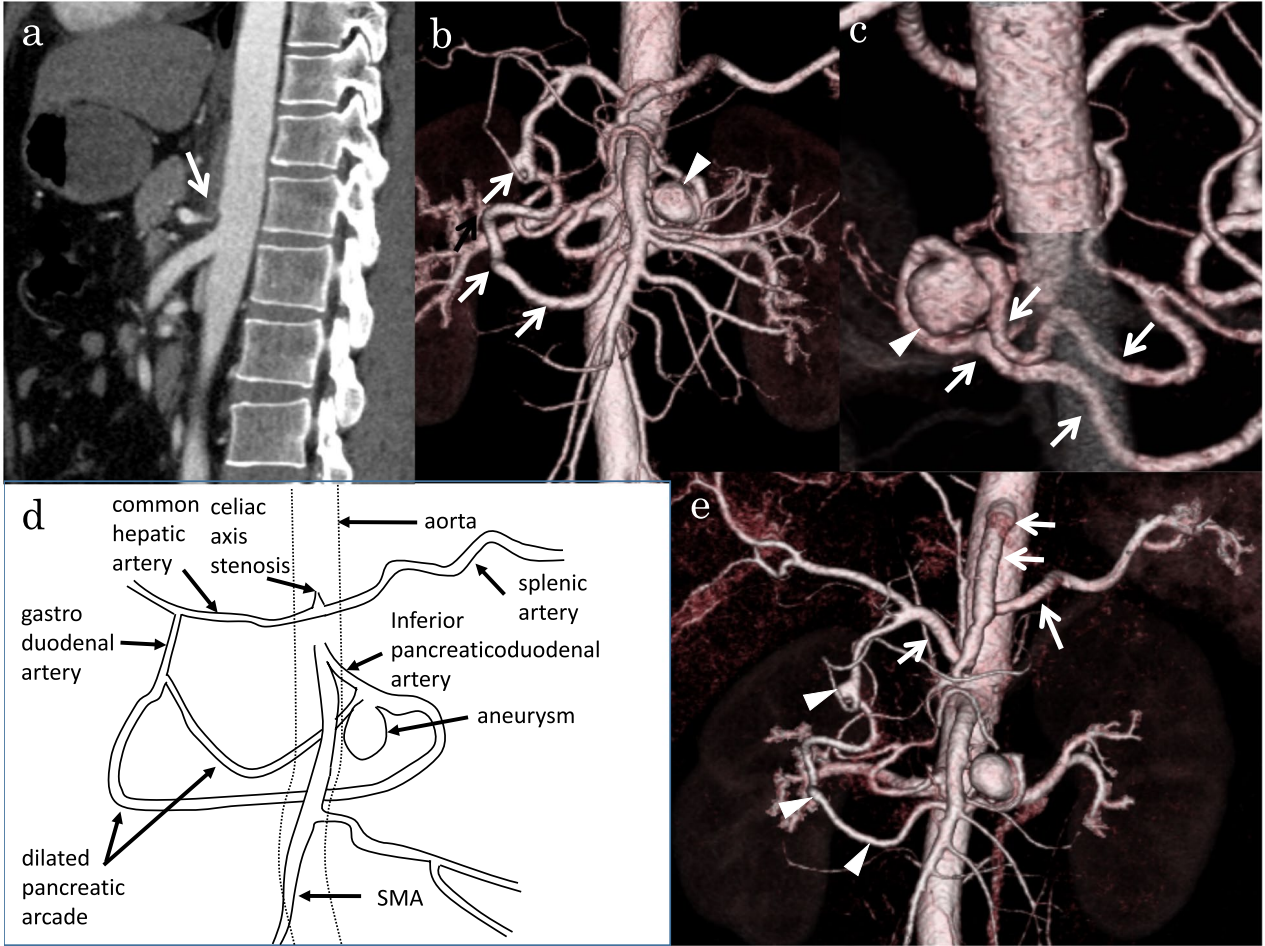
Images from Case 2. **a** Computed tomogram (sagittal plane) showing severe stenosis of the celiac axis (white arrow). **b**, **c** Three-dimensional computed tomogram showing markedly dilated pancreatic arcade (white arrows) with a large saccular pancreaticoduodenal artery aneurysm (white arrowheads). **d** The schematic diagram of Case 2. **e** Three-dimensional computed tomogram obtained 2 days after bypass surgery showing excellent flow of the hepatic and splenic arteries via bypass (white arrows) and decreased thickness of the pancreatic arcade (white arrowheads)

A 39-year-old man who had been in good health was found to have a PDAA with celiac stenosis during a medical checkup. A CT scan and superior mesenteric arteriography showed severe celiac axis stenosis (Fig. 5a) and a markedly dilated pancreatic arcade with a large saccular PDAA at the bifurcation of the anterior and inferior branches of the PDA (Fig. 5b–d). The long diameters of the aneurysm were 23 mm. We could not perform angiography of celiac artery because of celiac axis stenosis. The celiac axis stenosis was speculated to have occurred secondary to compression by the MAL. As in Case 1, TAE of PDAA cannot secure blood flow in the celiac artery system, we carried out elective surgery to release the MAL along with aortosplenic bypass.

**Fig. 6:**
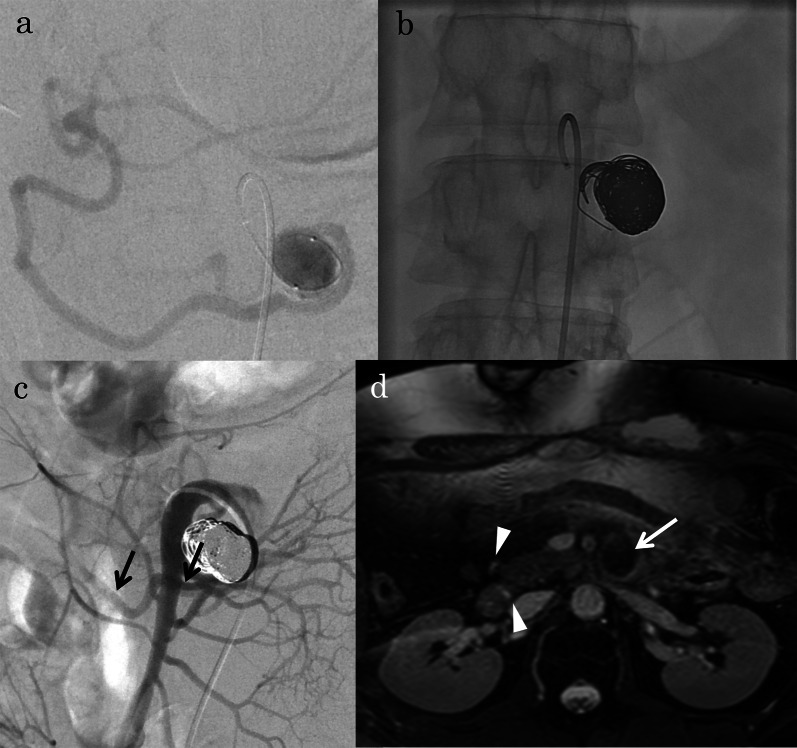
Images from Case 2. **a** Intra-aneurysmal angiogram immediately before coil embolization showing opacification of the distal inferior pancreaticoduodenal artery (PDA). **b** Fluoroscopic images during coil embolization showing coil packing of the inferior PDA aneurysm (PDAA). **c** Post-embolization angiogram via the superior mesenteric artery showing complete occlusion of the aneurysm with excellent preservation of the inferior PDA (black arrows). **d** Non-contrast-enhanced magnetic resonance imaging after treatment showing disappearance of the flow in the PDAA (white arrow) and decreased thickness of the pancreatic arcades (white arrowheads)

At 9 days postoperatively, antegrade flow of the hepatic and splenic arteries via bypass and decreased retrograde flow of the PDA via the SMA were confirmed by CT (Fig. 5e) and angiography (not shown). Therefore, coil packing of the aneurysm was performed (Fig. 6a, b). In total, eight detachable and three pushable microcoils were used. Post-embolization angiography showed complete occlusion of the aneurysm with excellent preservation of the inferior PDA (Fig. 6c). He developed no transient duodenal ischemia after TAE. He was administered 100 mg of aspirin daily after surgery and was discharged in good condition on postoperative day 17. At the 6-year 7-month follow-up observation, no recurrent perfusion or rupture of the PDAA had occurred (Fig. 6d), and the bypass graft showed excellent patency.

## Discussion

PDAAs are very rare, accounting for only 2% of all visceral aneurysms [1]. Unlike true aneurysms of the splenic artery, which rarely rupture when they are < 2 cm, PDAAs show no clear correlation between their size and potential for rupture [2]. Therefore, prompt intervention/treatment of all PDAAs is recommended at the time of diagnosis, irrespective of the size of the aneurysm.

The most frequent cause of PDAAs is pancreatitis (31%), followed by celiac axis stenosis (27%), atherosclerosis (24%), congenital disorders (13%), trauma (4%), and mycotic infections (4%) [3]. In one study, 24 (50%) of 48 PDAAs were caused by celiac axis stenosis [4]. In another study, compression by the MAL was responsible for celiac axis stenosis in 8 (18.6%) of 43 cases [5]. The cause of the PDAAs in our two cases was speculated to be celiac axis occlusion or severe stenosis due to compression by the MAL because neither patient had a history of pancreatitis, severe atherosclerosis, or trauma.

The therapeutic strategies for PDAAs involving the MAL must include both securement of the blood flow of the celiac arterial system and treatment of the aneurysm. The treatment methods used to secure the blood flow include both surgical procedures (MAL release and/or bypass surgery) and endovascular therapy (balloon dilation and/or celiac stenting) [4–12]. Treatment depends on the degree of celiac axis stenosis and the location and shape of the aneurysm. There are some reported cases of aneurysm regression or stability after simple reconstruction of severe celiac stenosis/occlusion [6, 7]. This suggests that decreasing the flow through the peripancreatic arteries by releasing the celiac axis stenosis and revascularizing the celiac territory could stabilize or decrease the size of the PDAA. However, some reports recommend additional treatments for the PDAA itself because of the risk of rupture [4, 5, 8, 9].

Eight cases of combined bypass surgery and TAE for PDAAs have been reported to date [4, 9–12]. The characteristics of these previous cases are summarized in Table 1. All TAE procedures were performed with coils using a packing technique. Among the eight patients, five and three had occlusion and stenosis by the MAL, respectively. Three patients developed rupture of the PDAA, and the remaining five did not. The optimal order of and duration of time between the two procedures (bypass surgery and coil embolization) have not yet been established. Two-stage treatment, which was applied in our two cases, was performed in six of the eight previously reported cases [4, 9, 11, 12]. Theoretically, for a patient with a ruptured PDAA, one-stage treatment (emergent TAE combined with simultaneous bypass operation) might be necessary to attain rapid hemostasis and sufficient blood flow of upper abdominal organs such as the liver. However, two-stage treatments were conducted even for ruptured PDAAs in two patients with stenosis by the MAL (TAE followed by bypass surgery) [4] and in one patient with occlusion by the MAL (bypass surgery followed by TAE) [9]. Thus, either one- or two-stage treatment can be an option depending on the patient’s condition, extent of stenosis (severe stenosis or occlusion), operator’s preference, or availability of a hybrid operation room in the clinical setting. In our cases, emergent treatments were not necessary because the PDAAs did not rupture. However, PDAAs involving the main trunk of the PDA are associated with celiac axis occlusion or severe stenosis. Therefore, occlusion by coil embolization would induce a high risk of loss of the major arterial supply of the liver and spleen. Hence, bypass surgery prior to coil embolization was first conducted to restore the normal arterial flow and prevent ischemia of those organs, followed by coil embolization at a 6- or 9-day interval. Saphenous vein graft was selected in 2 cases and expanded polytetrafluoroethylene graft was selected in 1 case and Dacron graft was selected in 4 cases, including our cases. Selection of the graft depends on several factors, such as the flow demand, the graft patency, the location and distance of vessels, resistance to infection, and patient status. Vascular prosthesis is better in the flow demand, but great saphenous vein is better in resistance to infection. In our cases, we selected Dacron graft to meet the flow demand adequately. Although the mean follow-up duration of eight patients was 40.8 months, our two cases were followed for relatively long period as 102 and 79 months without any recurrent perfusion and rupture. The combination of aortic bypass surgery and TAE for PDAA may have long-term outcome.

**Table 1:** Reported cases of pancreaticoduodenal artery aneurysm treated with bypass surgery and endovascular therapy

Patient	Authors	Year	Age (years)/sex	Aneurysm status/diameter	Celiac axis status	Treatment	Bypass	Graft	TAE	Interval between two treatments	Follow-up (months)
1	Bageacu et al. [4]	2006	55/unknown	Ruptured (15 mm)	Stenosis	TAE → bypass/two-staged	Aorto-to-hepatic artery	Unknown	Coil embolization	6 or 8 weeks	89
2			43/unknown	Ruptured (20 mm)	Stenosis	TAE → bypass/two-staged	Aorto-to-hepatic artery	Unknown	Coil embolization	6 or 8 weeks	78
3			51/unknown	Unruptured (18 mm)	Occlusion	Bypass → TAE/two-staged	Aorto-to-hepatic artery	Unknown	Coil embolization	Unknown	27
4	Teng et al. [9]	2006	46/M	Ruptured (19 mm)	Occlusion	Bypass → TAE/two-staged	Aorto-to-hepatic artery and SMA	Dacron	Coil embolization	1 day	unknown
5	Imamura et al. [10]	2011	61/M	Unruptured (20 mm)	Stenosis	Bypass → TAE/one-staged	Aorto-to-hepatic artery	SVG	Coil embolization	The same day	unknown
6	Nakano et al. [11]	2014	47/M	Unruptured (35, 15 mm)	Occlusion	Bypass → TAE/two-staged	Renal-to-splenic artery	ePTFE	Coil embolization	7 days	21
7	Simon et al. [12]	2017	39/F	Unruptured (40 mm)	Occlusion	TAE → bypass/one-staged	Aorto-to-hepatic artery	SVG	Coil embolization	The same day	12
8			61/F	Unruptured (20 mm)	Occlusion	Bypass → TAE/two-staged	Aorto-to-celiac artery	Dacron	Coil embolization	1 month	18
9	Current report	2021	54/F	Unruptured (21, 19 mm)	Occlusion	Bypass → TAE/two-staged	Aorto-to-splenic artery	Dacron	Coil embolization	6 days	102
10			39/M	Unruptured (23 mm)	Stenosis	Bypass → TAE/two-staged	Aorto-to-splenic artery	Dacron	Coil embolization	9 days	79

*TAE* transcatheter arterial embolization, *SMA* superior mesenteric artery, *SVG* saphenous vein graft, *ePTFE* expanded polytetrafluoroethylene

## Conclusion

Combined treatment with bypass surgery and coil embolization can be an effective option for the treatment of PDAAs associated with celiac axis occlusion or severe stenosis. Further follow-up is necessary to clarify the efficacy of our combination treatment in terms of prevention of PDAA rupture and long-term patency of the bypass graft.

## Data Availability

Not applicable.

## Abbreviations

PDAA: Pancreaticoduodenal artery aneurysm
MAL: Median arcuate ligament
CT: Computed tomography
TAE: Transcatheter arterial embolization
PDA: Pancreaticoduodenal artery
SVG: Saphenous vein graft
ePTFE: Expanded polytetrafluoroethylene
